# New reference values for ultrasound fetal biometry in Japanese population and comparison with other studies

**DOI:** 10.1038/s41598-025-14508-9

**Published:** 2025-08-20

**Authors:** Sumito Nagasaki, Keisuke Ishii, Yoshitaka Murakami, Anna Tsutsui, Nobuhiro Hidaka, Hironori Takahashi, Kiyotake Ichizuka, Kei Miyakoshi, Kiyonori Miura, Susumu Miyashita, Yoshimasa Kamei, Masahiko Nakata, Study Group on New Reference Values for Fetal Biometry in Japan, Sumito Nagasaki, Yohei Onodera, Hiroaki Aoki, Atsuo Kiuchi, Katsuhiko Naruse, Mariko Inoue, Ritsuko K. Pooh, Katsuhiko Naruse, Shigenori Iwagaki, Akihito Morita, Mariko Serizawa, Kotaro Iino, Satoru Ikenoue, Shigeru Inoue, Jun Yamauchi, Tomohiko Ishida, Kazuya Abe, Madoka Tsuzuki, Sumiko Hasegawa, Asuka Higuchi, Mariko Tomosaka, Momo Kawanami, Ikumo Tanaka, Masamichi Hoshino, Hirotada Suzuki, Manabu Ogoyama, Kenji Horie, Kayo Takahashi, Tomoyuki Kuwata, Yuka Yamamoto, Nana Matsuzawa, Koyo Yoshida, Naoko Suga, Ai Takamizu, Akari Koizumi, Kenji Kanenishi, Yasuyuki Fujita, Ryo Kiyokoba, Hiroshi Masaoka, Hideo Matsuda, Maki Sekihara, Atsushi Yoshida, Shintaro Maki, Tomoaki Ikeda, Kayo Tanaka, Hiroaki Tanaka, Eiji Kondo, Masafumi Nii, Shoichi Magawa, Sho Takakura, Mizuki Yamaguchi, Mikiko Nishioka, Toshiyuki Hata, Aya Koyanagi, Ken Miyazaki, Katsusuke Ozawa, Rika Sugibayashi, Seiji Wada, Jin Muromoto, Yuri Hasegawa, Naoki Okimoto, Kazufumi Haino, Shoji Satoh, Naoko Koyama, Hisashi Masuyama, Eriko Eto, Akiko Ohira, Kazumasa Tani, Yoko Nagayasu, Tetsu Wakimoto, Akihiko Kikuchi, Sachi Kijima, Shunsuke Tamaru, Shigeru Itoh, Saori Tanaka, Masayuki Someya, Takeshi Murakoshi, Hiroko Konno, Norihiko Kikuchi, Ryu Matsuoka, Yuka Yamashita, Maya Machi, Katsufumi Otsuki, Reina Komatsu, Minako Goto, Ayumi Okuyama, Junichi Hasegawa, Natsumi Furuya, Chika Homma, Hajime Ota, Takashi Yoshimasu, Eijiro Hayata, Makiko Shimabukuro, Hikari Kotaki, Mayumi Takano, Misako Iwata, Rie Oi, Mao Sakura, Arihiro Shiozaki, Akitoshi Nakashima, Emi Kondo, Hidetaka Hayashi, Hirokazu Yorioka

**Affiliations:** 1 Department of Obstetrics and Gynecology, Toho University Omori Medical Center, 6-11-1, Omorinishi, Ota-ku, Tokyo 143-8540 Japan; 2 Department of Maternal Fetal Medicine, Osaka Women’s and Children’s Hospital, 840, Murodo-cho , Izumi-shi, Osaka 594-1101 Japan; 3 Department of Medical Statistics, Toho University, 5-21-16, Omorinishi, Ota-ku, Tokyo 143-8540 Japan; 4 Department of Obstetrics, Fukuoka Children’s Hospital, 5-1-1, Kashiiteriha, Higashi-ku, Fukuoka-shi, Fukuoka 813-0017 Japan; 5 Department of Obstetrics and Gynecology, Jichi Medical University Hospital, 3311-1, Yakushiji, Shimotsuke-shi, Tochigi 329-0498 Japan; 6 Department of Obstetrics and Gynecology, Showa Medical University Northern Yokohama Hospital, 35-1, Chigasakichuo, Tuzuki-ku, Yokohama-shi, Kanagawa 224-8503 Japan; 7 Department of Obstetrics and Gynecology, International Catholic Hospital, 2-5-1, Nakaochiai, Shinjuku-ku, Tokyo 161-8521 Japan; 8 Department of Obstetrics and Gynecology, Nagasaki University Graduate School of Biomedical Sciences, 1-12-4, Sakamoto, Nagasaki-shi, Nagasaki 852-8523 Japan; 9 Department of Maternal Fetal Medicine, Miyagi Children’s Hospital, 4-3-17, Ochiai, Aoba-ku, Sendai-shi, Miyagi 989-3126 Japan; 10 Department of Obstetrics and Gynecology, Saitama Medical University Hospital, 38, Morohongo, Moroyama-cho, Iruma-gun, Saitama 350-0495 Japan; 11 Akita University Hospital, Akita, Japan; 12 Aoki Obstetrics and Gynecology Clinic, Tokyo, Japan; 13 ARTEMIS Utsunomiya Clinic, Tochigi, Japan; 14 Chiba Kaihin Municipal Hospital, Chiba, Japan; 15 CRIFM Prenatal Medical Clinic, Osaka, Japan; 16 Dokkyo Medical University Hospital, Tochigi, Japan; 17 Gifu Prefectural General Medical Center, Gifu, Japan; 18 Gunma University Hospital, Gunma, Japan; 19 Hamamatsu Medical Center, Shizuoka, Japan; 20 Iino Hospital, Tokyo, Japan; 21 Inoue Ladies’ Clinic, Kumamoto, Japan; 22 Itabashi Chuo Medical Center, Tokyo, Japan; 23 Iwajuku Clinic, Gunma, Japan; 24 Jichi Medical University Saitama Medical Center, Saitama, Japan; 25 Juntendo University Hospital, Tokyo, Japan; 26 Juntendo University Urayasu Hospital, Chiba, Japan; 27 Kagawa University Hospital, Kagawa, Japan; 28 Kyushu University Hospital, Fukuoka, Japan; 29 Masaoka Hospital, Hiroshima, Japan; 30 Matsuda perinatal clinic, Saitama, Japan; 31 Medical Park Iruma, Saitama, Japan; 32 Mie University Hospital, Mie, Japan; 33 Miyake Clinic, Okayama, Japan; 34 Miyazaki Ladies Clinic, Mie, Japan; 35 National Center for Child Health and Development, Tokyo, Japan; 36 NHO Okayama Medical Center, Okayama, Japan; 37 Niigata University Medical & Dental Hospital, Niigata, Japan; 38 Oita Prefectural Hospital, Oita, Japan; 39 Okayama University Hospital, Okayama, Japan; 40 Osaka Medical and Pharmaceutical University Hospital, Osaka, Japan; 41 Saitama Medical Center, Saitama Medical University, Saitama, Japan; 42 Sakuradai Maternity Clinic, Tokyo, Japan; 43 San-Ikukai Hospital, Tokyo, Japan; 44 Sappro Medical University Hospital, Hokkaido, Japan; 45 Seirei Hamamatsu General Hospital, Shizuoka, Japan; 46 Shinshu University Hospital, Nagano, Japan; 47 Showa Medical University Hospital, Tokyo, Japan; 48 Showa Medical University Koto Toyosu Hospital, Tokyo, Japan; 49 St. Marianna University Hospital, Kanagawa, Japan; 50 Teine Keijinkai Hospital, Hokkaido, Japan; 51 Tokyo Metropolitan Ohtsuka Hospital, Tokyo, Japan; 52 Toyama University Hospital, Toyama, Japan; 53 University of Occupational and Environmental Health, Kitakyushu, Fukuoka Japan; 54 Yakumaru Hospital, Chiba, Japan; 55 Yorioka Fetus Clinic, Osaka, Japan

**Keywords:** Estimated fetal weight, Reference values, Ultrasonography, Japanese population, Fetal Biometry, Ultrasonography, Medical research, Epidemiology

## Abstract

Given the decline in birthweights over the past 30 years in Japan and advancements in ultrasound technology, this study aimed to establish new reference values for ultrasound fetal biometry in Japan and to compare them with international and Asian studies. We conducted a cross-sectional prospective study involving singleton pregnancies who received prenatal checkups at obstetric facilities across Japan. During routine prenatal care, ultrasound measurements—biparietal diameter (BPD), head circumference (HC), abdominal circumference (AC), and femur length (FL)—were recorded. Estimated fetal weight (EFW) was calculated using both the Shinozuka formula, commonly used in Japan, and the Hadlock-3 formula, widely used internationally. Using the collected data, we developed gestational-age-specific reference values for BPD, HC, AC, FL, and EFW by applying best-fitted fractional polynomial regression, and compared them with existing international standards and reference values from other Asian countries. The mean EFW calculated by the Hadlock formula in this study tended to be smaller than that reported in other international studies, and BPD, HC, AC, and FL were also generally smaller than those observed in other Asian references. These findings indicate that fetal biometry values in the Japanese population are smaller not only than international standards but also compared with those from other Asian countries, and suggest that these population-specific data may contribute to improving the accuracy of fetal growth assessment in Japan and other parts of Asia.

## Introduction

Fetal growth assessment is essential for obstetric management and the use of appropriate reference or standard for fetal biometry is necessary. Large-scale international studies, such as the World Health Organization (WHO)^1^ and INTERGROWTH-21 studies^2–4^ have been published and widely used. However, the application of universal standard values requires careful consideration since fetal biometry is influenced by ethnic, regional and temporal differences. Asians tend to have smaller fetal biometry than other races^5^. This suggests that fetal biometry in Japanese population should be assessed using their own biometric charts. We in Japan have employed standard values published by the Japan Society of Ultrasonics in Medicine (JSUM) in 2003^6^, which was based on data from 1985 to 1995^7^. On the other hand, birthweights at term have increased globally^8^ but, in Japan over the past 30 years birthweights have declined and the proportion of low birthweights were increasing for the last 30 years^9–11^. Furthermore, there have also been considerable advancements in ultrasound technology in few decades. Thus, reassessment of fetal biometry charts might be needed. With this background, we reestablished new percentile chart for ultrasound fetal biometry using a cross-sectional study in a Japanese population. While the debate on whether reference or standard charts are more appropriate for fetal growth assessment remains ongoing^12,13^, many national or local charts have adopted the reference approach, whereas broader international projects such as INTERGROWTH-21st and WHO have used standard-based methods. Based on this context, we adopted the reference approach in the present study to reflect fetal growth patterns in the general Japanese population. Additionally, we compared our results with other globally published studies.

## Methods

### Study design

This cross-sectional prospective study involved singleton pregnancies who underwent prenatal checkups at obstetric facilities across Japan. In this study, ultrasound data were collected as part of a research-specific protocol designed to ensure high data quality. We obtained the data of fetal biometry and perinatal outcomes from each participant. Regarding fetal biometry, one measurement for each patient was used for the analysis during the index pregnancy. Maternal background (age at delivery, pre-pregnancy weight, height, conception method, the presence of hypertensive disorders of pregnancy (HDP), and hyperglycemic disorders in pregnancy (HIP)) and information on newborns (gestational age at delivery, sex, and birthweight) were collected. HDP and HIP were diagnosed using the Guideline for Obstetrical Practice in Japan^14^. Specifically, HDP were classified into the four categories: preeclampsia, gestational hypertension, superimposed preeclampsia, and chronic hypertension. HIP include diabetes before pregnancy, overt diabetes in pregnancy and gestational diabetes mellitus (GDM). Obstetrical management for pregnancy-related complications including HDP and HIP was conducted based on the clinical guideline from Japan Society of Obstetrics and Gynecolog^14^.

### Settings

For building a reference from unselected populations, participating facilities across Japan were publicly recruited through the JSUM’s mailing list (The number of facilities registered on the mailing list was 134). The enrollment period was from August 2022 to September 2022. Data collection was carried out from March 2023 through September 2024. The ultrasound devices used in this study were those manufactured by GE HealthCare (Chicago, IL, USA), Canon Medical Systems (Tochigi, Japan), Philips Healthcare (Eindhoven, Netherlands), Samsung Medison (Seoul, South Korea), and Hitachi (FUJIFILM) (Tokyo, Japan), which are commonly used in Japan. Transvaginal or convex probes with transmitting frequency of 1-10MHz were used in each facility. This study was registered with the University Hospital Medical Information Network (UMIN) (UMIN Study ID: UMIN000050614).

### Study population

We included singleton pregnancies who were taken care at participating facilities during the study period. In Japan, there are no recommended gestational ages for systematic ultrasound examinations during pregnancy, allowing measurements to be performed at any gestational age. In this study, pregnant women were recruited at the time of their routine prenatal check-ups after the initiation of the study. Eligibility criteria for participants were presence of the cross-section of ultrasound images enabling the measurement of biparietal diameter (BPD), head circumference (HC), abdominal circumference (AC), and femur length (FL). We did not exclude cases who met the eligibility criteria at the time of ultrasound measurement even if structural abnormalities were found after birth.

### Ultrasound measurement of fetal biometry

Gestational age was calculated based on the date of embryo transfer for pregnancies conceived by embryo transfer. For other pregnancies, gestational age was calculated from the last menstrual period (LMP) and confirmed by an accurately measured crown-rump length (CRL) during the first trimester. If there was a discrepancy of more than seven days between the LMP-based and ultrasound-based estimates, gestational age was adjusted according to the CRL measurement^14^. Ultrasound measurements of fetal biometry were conducted according to established standard techniques^7,15^. Ultrasound measurements of BPD were performed from 11 to 41 weeks of gestation, while HC, AC, and FL were measured from 16 to 41 weeks of gestation.

### Sample size

Sample size was set at 3000 cases with reference to the study by Altman et al.^16^ Therefore, the target number of cases to be collected per facility was set at about 60.

### Prevention of biases

To minimize bias in this study, we referred to the systematic review by loannou et al^17^. To avoid including an excessive number of high-risk pregnancies and to approximate a population-based study, we excluded women who were transferred to tertiary care center unit for medical reasons after the first trimester, as well as those deemed unsuitable for enrollment for non-medical reasons. Furthermore, to ensure the quality of ultrasound measurements, participating facilities were limited to those staffed by board-certified fellows of the JSUM, ensuring that ultrasound examination was performed under the guidance of the specialists. Ultrasound measurements were taken consecutively and non-selectively after the start of the study.

### Statistical analysis

We analyzed BPD, HC, AC, FL and EFW calculated using BPD, HC, AC, and FL. EFW was calculated using formulas proposed by Shinozuka^18–20^ and Hadlock^21^. The actual formula used in this study were follows; Shinozuka formula, which is mainly used in Japan: EFW = 1.07 × BPD^3^ + 0.3 × AC^2^ × FL” and Hadlock-3 formula, which is commonly used other countries: log _10_(EFW) = 1.326 - 0.00326 × AC × FL + 0.0107 × HC + 0.0438 × AC + 0.158 × FL” which is commonly used other countries. Statistical analysis was conducted according to previous reports on fetal biometry measured using ultrasound during pregnancy^22–26^. A regression model was built, where BPD, HC, AC, FL or EFW were determined to be the response variable (Y) and gestational age to be the predictor variable (X). We calculated the 3rd, 10th, 50th, 90th, and 97th percentiles, as well as the SD value on this regression model. First, we described the correlation plots of the gestational age and the endpoints and checked the symmetry of marginal distribution of each gestational age. The symmetry of distribution enabled us to assume the marginal distribution was normal. Second, a fractional polynomial regression was applied to describe the relationship between gestational age and each fetal biometry. For likelihood ratio tests, the best-fit model for each measurement was selected. Since the residual was dependent on gestational age, a polynomial regression was performed between the absolute residual and gestational age, and the fitted value of the model was multiplied by $\sqrt{\frac{\uppi }{2}}$ (approximately 1.253) to yield gestational age-specific SD. This is because the parametric method of deriving a reference range assumes that the variable follows a normal distribution at all gestational age. Consequently, the residuals at each gestational age should also follow a normal distribution, and their absolute values should follow a half-normal distribution. The mean of a half standard normal distribution is known to be $\sqrt{\frac{\uppi }{2}}$. Thus, the mean of the absolute residuals multiplied by $\sqrt{\frac{\uppi }{2}} provides$ an estimate of the SD of the residuals^27^. Finally, the 10^th^ and 90^th^ percentiles were calculated as mean ± 1.28 × SD, and the 3^rd^ and 97^th^ percentiles were calculated as mean ± 1.88 × SD.

The comparison with large-scale international standards was performed using the mean EFW calculated by the Hadlock formula. The formula to calculate Z-scores for the comparisons with other Asian countries^28–36^ was as follows: $\text{Z}=\frac{{\text{X}}_{\text{GA}} {-\text{ M}}_{\text{GA}}}{{\text{SD}}_{\text{GA}}}$, where X_GA_ is the data from other populations at a known gestational age, M_GA_ is the mean value of this study, and SD_GA_ is the SD of this study. Z-score close to zero indicated that there was no difference in values between the two populations, and Z-score greater than zero indicated that the mean value of the Japanese population data in this study was smaller than the mean value of the other Asian populations. Z-score in the range of ± 1 indicated a difference of ± 1 SD. All analyses were performed using STATA version 18.0 (Stata Corp. TX, USA).

### Ethical considerations

This study was approved by the Ethics Committee of Toho University Faculty of Medicine (Approval Nos. A23056_A23026_A22069). The requirement for written informed consent was formally waived by the Ethics Committee of Toho University Faculty of Medicine, as the study used only patients’ data. An opt-out process was employed, and information about the study was made available at each participating institution to allow patients the opportunity to decline participation. The study was conducted in accordance with the principles of the Declaration of Helsinki.

## Results

### Participants

Fifty-four facilities in all seven regions across Japan (Hokkaido, Tohoku, Kanto, Chubu, Kinki, Chugoku, and Kyushu/Okinawa) participated in this study. The number of measurements per facility was 61(IQR 57—62). Overall, 3,753 participants, including 1,369 from primary medical care facilities and 2,384 from tertiary perinatal centers were included.

### Data description

A total of 3,753 measurements were available for BPD, 2,787 for HC, 2,939 for AC, and 2,947 for FL. EFW was calculated for 2,939 cases using the Shinozuka formula and for 2,779 cases using the Hadlock formula. Table 1 showed the Maternal characteristics and pregnancy and neonatal outcomes in study population. The median maternal age at delivery was 34years. The maternal height was 158 cm, and the maternal weight before pregnancy was 53.0kg. The gestational age at delivery was 39.1weeks, and the birthweight was 3,000g. The proportion of HDP and HIP was 6.3% and 7.5%, respectively.

**Table 1:** Clinical characteristics and outcomes.

Parameter	Value
Maternal age at delivery (years)	34 (30.0–38.0)
Maternal height (cm)	158 (155–162)
Maternal BW before pregnancy (kg)	53.0 (48.0–59.0)
ART	861 (22.9%)
Gestational age at delivery (weeks)	39.1 (38.1–40.1)
Birthweight (g)	3,000 (2,738–3,264)
Sex	
Male	1,906 (50.8%)
Female	1,826 (48.7%)
No data	21 (0.6%)
Hypertensive disorders of pregnancy	236 (6.3%)
Gestational hypertension	92 (2.5%)
Preeclampsia	87 (2.3%)
Superimposed preeclampsia	15 (0.4%)
Chronic hypertension	42 (1.1%)
None	3,494 (93.1%)
No data	23 (0.6%)
Hyperglycemic disorders in pregnancy	282 (7.5%)
Gestational diabetes mellitus	245 (6.5%)
Overt diabetes in pregnancy	7 (0.2%)
Diabetes before pregnancy	30 (0.8%)
None	3,449 (91.9%)
No data	22 (0.6%)

Data are provided as median (interquartile range) or n (%).

ART, assisted reproductive technology; BW, body weight.

## Main results

### Reference values for ultrasound fetal biometry

**Fig. 1:**
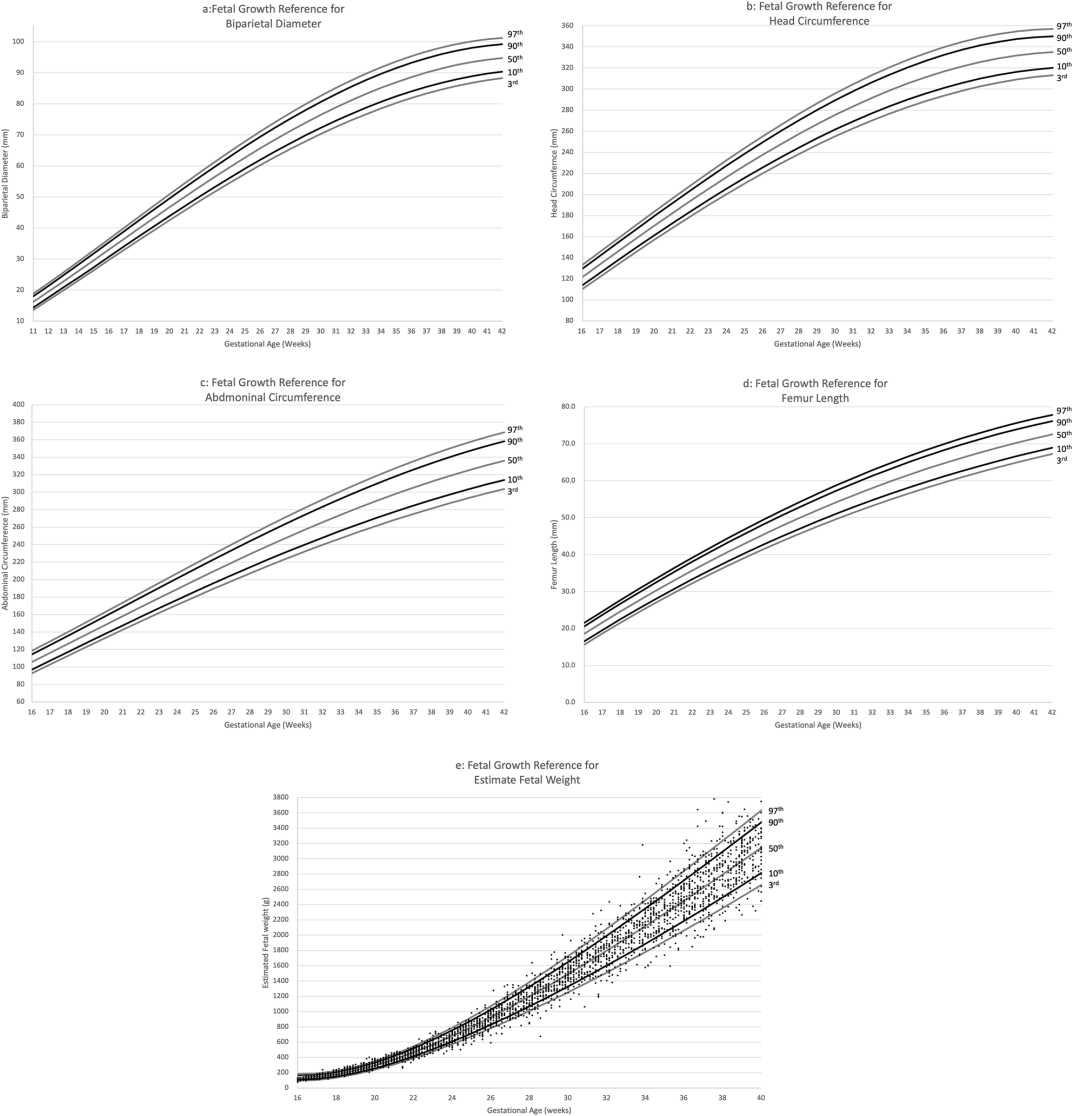
Smoothed centile curve and scatter plot for fetal biometry according to gestational age. The following fitted centiles according to gestational age are presented from bottom to top at 3^rd^, 10^th^, 50^th^, 90^th^,97^th^. biparietal diameter (**a**); head circumference (**b**); abdominal circumference (**c**); femur length (**d**); estimate fetal weight (**e**).

Table 2 showed the corresponding values of EFW at each gestational age. After 20 weeks of gestation, EFW calculated using the Shinozuka’s formula tended to be larger than that calculated using the Hadlock-3 formula. Figure 1a-e showed the 3rd, 10th, 50th, 90th, and 97th percentiles at each gestational age of BPD, HC, AC, FL and EFW(Shinozuka) respectively. Table S1-S4 (Supplementary Information 1) showed the corresponding values and their SD of the BPD, HC, AC, FL at each gestational age. Table 3 showed the formulas for estimating the mean and SD of EFW, BPD, HC, AC, and FL from each gestational age.

**Table 2:** Mean and SD of the 3rd, 10th, 90th, and 97th percentiles of EFW calculated using the Shinozuka’s and Hadlock-3 formulas.

	EFW by Shinozuka’s formula						EFW by Hadlock-3 formula					
GA	3rd	10th	50th	90th	97th	SD	3rd	10th	50th	90th	97th	SD
16	85	102	139	176	194	29.0	110	132	179	226	248	36.8
17	95	111	147	183	200	28.0	124	143	182	221	239	30.5
18	123	141	178	216	233	29.2	151	167	202	237	253	27.1
19	167	187	228	269	289	32.2	189	205	239	273	289	26.7
20	224	247	294	341	364	37.0	236	253	290	327	344	28.8
21	292	318	374	430	456	43.5	291	311	354	397	417	33.5
22	369	400	466	532	563	51.6	354	378	430	482	506	40.4
23	453	490	568	646	683	61.1	424	453	517	580	609	49.3
24	544	587	679	771	814	71.9	500	536	613	690	726	60.1
25	641	691	799	906	956	83.9	581	625	718	811	854	72.6
26	743	801	925	1049	1107	97.0	669	720	831	942	994	86.5
27	849	916	1058	1200	1266	111.0	761	822	952	1082	1143	101.7
28	960	1035	1196	1357	1433	125.8	858	929	1080	1231	1302	118.0
29	1074	1159	1340	1521	1605	141.4	960	1041	1214	1387	1468	135.1
30	1191	1286	1487	1689	1784	157.5	1067	1158	1354	1549	1641	152.8
31	1312	1417	1640	1862	1967	174.1	1178	1280	1499	1718	1821	171.0
32	1436	1551	1795	2040	2155	191.1	1294	1407	1650	1892	2006	189.5
33	1563	1688	1955	2221	2346	208.3	1414	1539	1805	2071	2196	208.0
34	1693	1828	2117	2405	2541	225.5	1539	1675	1965	2255	2391	226.4
35	1825	1971	2282	2593	2738	242.8	1669	1816	2129	2442	2588	244.4
36	1961	2117	2449	2782	2938	259.9	1804	1961	2297	2632	2789	261.9
37	2099	2265	2620	2974	3140	276.7	1944	2111	2468	2825	2992	278.6
38	2240	2416	2792	3167	3343	293.2	2089	2266	2643	3020	3196	294.4
39	2385	2570	2966	3362	3547	309.2	2239	2425	2821	3216	3402	309.1
40	2532	2727	3142	3557	3752	324.5	2395	2589	3002	3414	3608	322.4
41	2682	2886	3320	3754	3957	339.1	2557	2758	3185	3613	3814	334.2
42	2836	3048	3499	3951	4163	352.8	2725	2931	3372	3812	4019	344.2

EFW, estimated fetal weight; SD, standard deviation.

**Table 3:** Equation and Adjusted R^2^ for Standard Deviations and mean of estimated fetal weight according to gestational age.

Mean	Equation (GA: exact weeks)	Adjusted R^2^
BPD (mm)	0.4529736 × GA^2^ - 0.1056133 × GA^2^ × log (GA) - 7.949686	0.9875
HC (mm)	1.660305 × GA^2^ - 0.3893129 × GA^2^ × log (GA) - 26.84611	0.9755
AC (mm)	1.209765 × GA^2^ - 0.2709246 × GA^2^ × log (GA) - 11.69583	0.9661
FL (mm)	4.333097 × GA - 0.0389381 × GA^2^ - 40.79377	0.9767
EFW Shinozuka (g)	55,319.19 × GA^−1^ + 211.5536 × GA - 6703.086	0.9662
EFW Hadlock 3 (g)	21,070.63 - 12,381.79 × GA^0.5^ + 2582.036 × GA^0.5^ × log (GA)	0.9621
SD	Equation (GA: exact weeks)	Adjusted R^2^
BPD (mm)	1.253 × (1.248556 - 0.085541 × GA + 0.008052 × GA^2^ − 0.000123 × GA^3^)	0.1110
HC (mm)	1.253 × (10.12058 - 0.9686606 × GA + 0.0507207 × GA^2^ - 0.0006693 × GA^3^)	0.0665
AC (mm)	1.253 × (12.33031 -1.144092 × GA + 0.0549079 × GA^2^ −0.0006393 × GA^3^)⇨1.253 × (12.33031 - 1.144092 × GA + 0.0549079 × GA^2^ − 0.0006393 × GA^3^)	0.1069
FL (mm)	1.253 × (2.454694 - 0.1983582 × GA + 0.0096214 × GA^2^ − 0.0001194 × GA^3^)	0.0496
EFW Shinozuka (g)	1.253 × (334.8826 - 41.51368 × GA + 1.634357 × GA^2^- 0.0160988 × GA^3^)	0.3287
EFW Hadlock 3 (g)	1.253 × (572.8827 - 68.37788 × GA + 2.576247 × GA^2^ − 0.0266007 × GA^3^)	0.3370

AC, Abdominal circumference; BPD, Biparietal diameter; EFW, estimated fetal weight; FL, Femur length; fetal weight; GA, gestational age; HC, Head circumference; SD, standard deviation.

### Comparison with other Studies

**Fig. 2:**
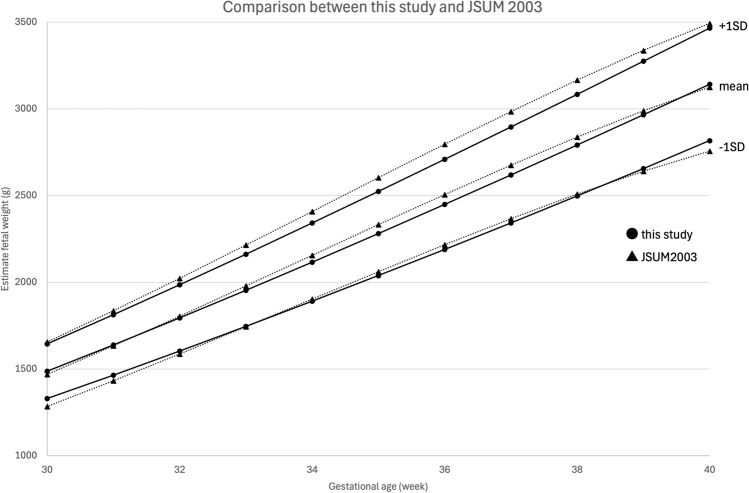
Comparison of the mean and SD of EFW between this study and JSUM2003.

**Fig. 3:**
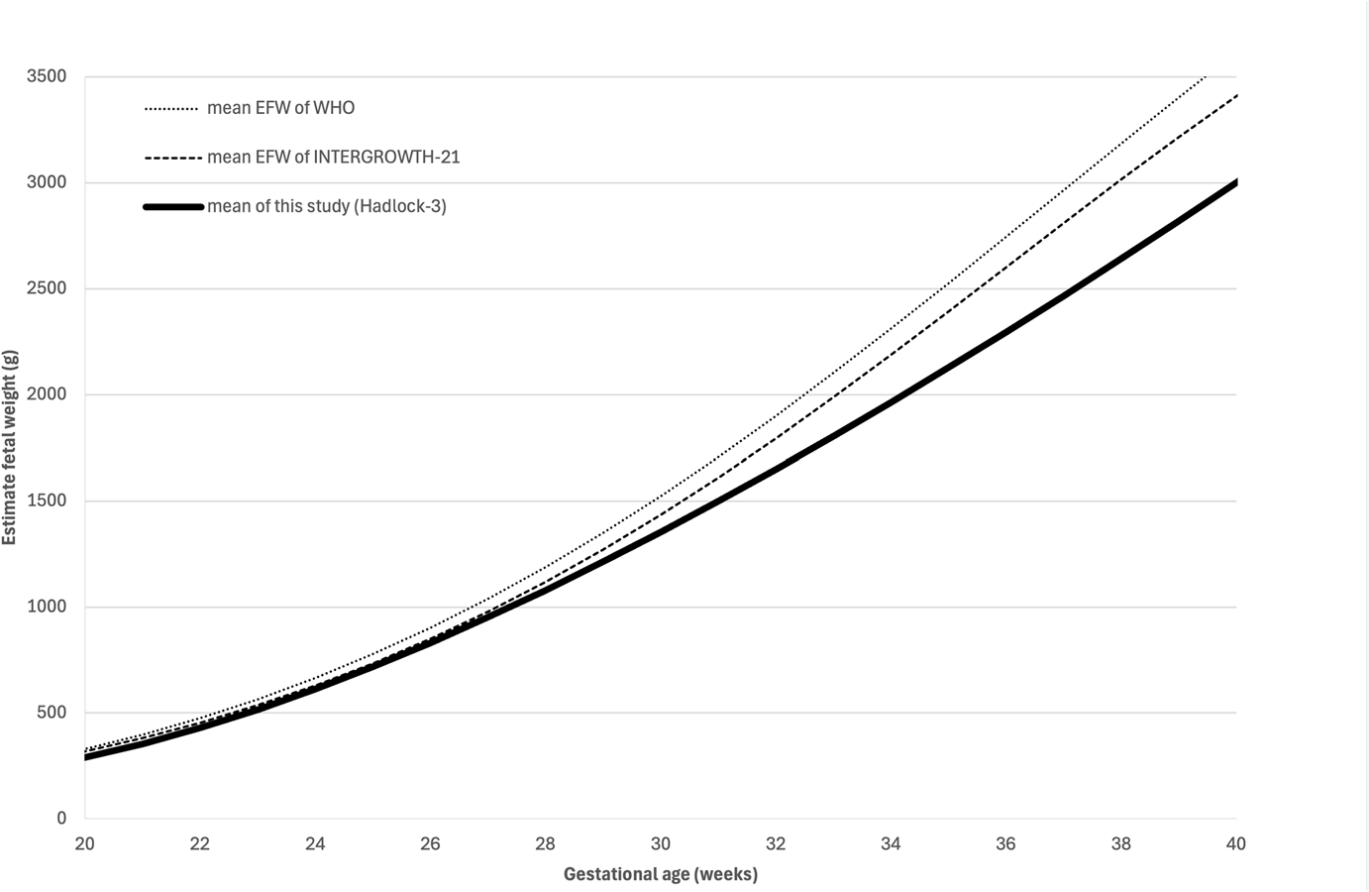
Comparison of the mean EFW between this study and international studies, including the INTERGROWTH-21 and WHO studies. Mean of EFW in WHO (dotted line); Mean of EFW in INTERGROWTH-21 (dashed line); Mean of EFW (continuous). EFW, estimated fetal weight; WHO, World Health Organization.

**Fig. 4:**
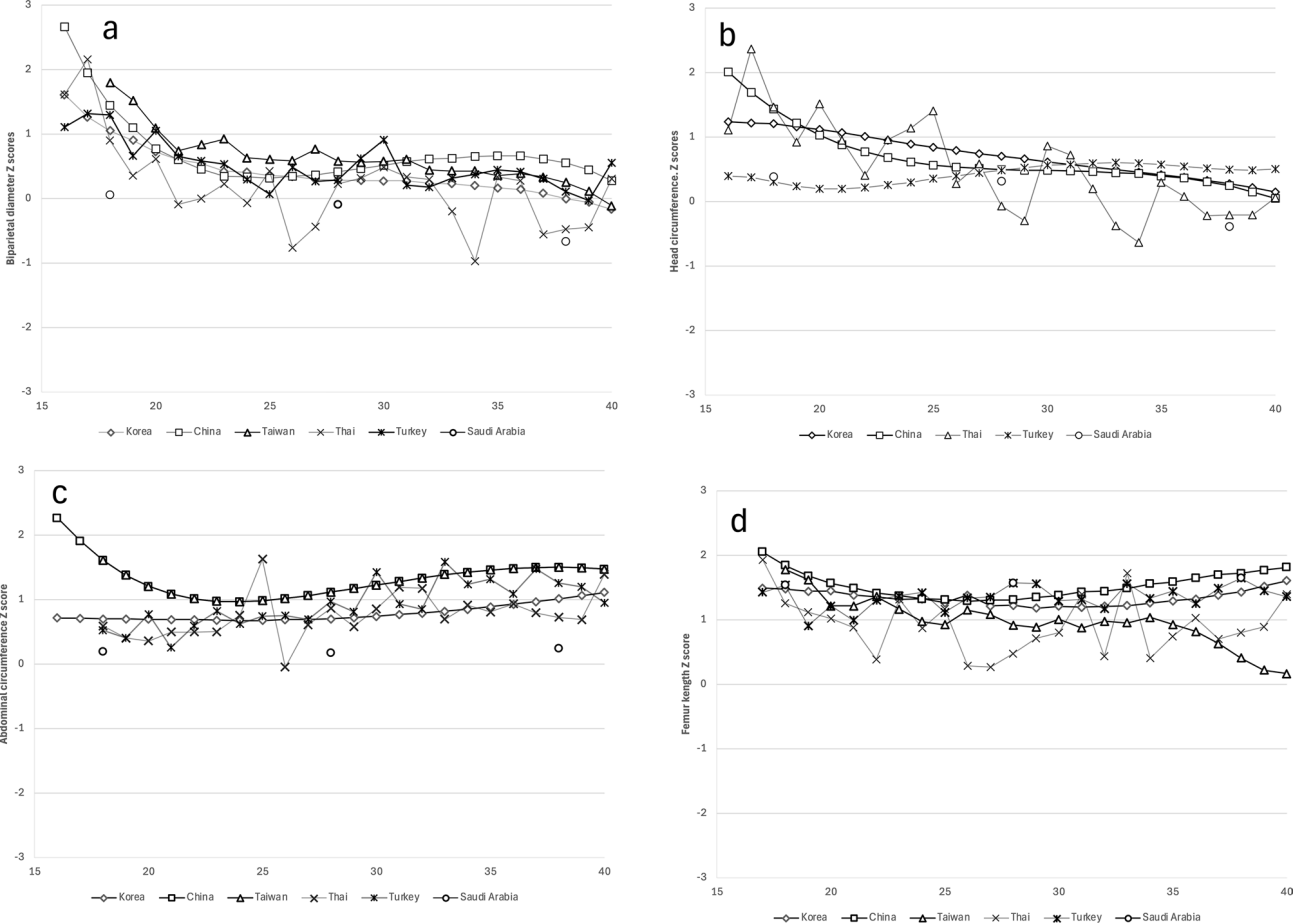
Comparison of Z-scores for biometric ultrasound parameters between Japan and other Asian countries. Comparison of Z-scores for biparietal diameter (**a**); Comparison of Z-scores for Head circumference (**b**); Comparison of Z-scores for Abdominal circumference (**c**); Comparison of Z-scores for Femur length (**d**). Z-scores for each parameter were calculated using reference values in other Asian countries. Korea (rhombus); China (square); Taiwan (triangle); Thai (cross); Turkey (asterisk); Saudi Arabia (circle).

Compared to the JSUM 2003 data, EFW in our study showed a tendency to be smaller, especially after 30 weeks of gestation (Fig. 2). And, the mean EFW calculated by Hadlock-3 formula tended to be smaller at all gestational age compared with the mean EFW reported in large scaled international WHO^1^ and INTERGROWTH-21^4^ studies (Fig. 3). Additionally, Ultrasound fetal biometry obtained in this study was also compared with published reference data from other Asian countries^28–36^ (Figs. 4a–d). The Z-scores for BPD, HC (excluding Taiwan due to lack of data), AC, and FL exceeded zero at almost all gestational ages, indicating that the dimensions of Japanese population tended to be smaller than those of other Asian populations.

## Discussion

We established new reference values for ultrasound fetal biometry in Japanese population using a prospective design with minimal bias, and compared these values with those reported in international studies. Furthermore, this is the first report to compare fetal ultrasound biometry among Asian countries. Although it is generally known that Asian populations tend to have smaller biometric measurements than Caucasians, our findings revealed that fetal biometry in Japan was smaller than that in other Asian countries.

### Comparison with other Studies

In Japan, the decline in birthweight has been reported over the past 30 years. Our findings are consistent with this trend, as EFW in our study population tend to be smaller than that reported in the JSUM 2003, particularly after 30 weeks of gestation (Fig. 2). Moreover, it has been previously reported^5^ that Asians are characterized by having small fetal biometric dimensions, the mean EFW calculated by Hadlock-3 formula in this study was smaller than that reported in the INTERGROWTH-21 [2] and WHO studies^1^ (Fig. 3). The INTERGROWTH-21 standards^2^ were developed based on data from Brazil, Italy, Oman, UK, USA, China, India, and Kenya, and the WHO standards were developed based on data from Argentina, Brazil, DR Congo, Denmark, Egypt, France, Germany, India, Norway, and Thailand. Furthermore, this study showed that fetuses in Japan were smaller than those in other Asian countries (Fig. 4a–d). A study using data on newborns in the USA reported that the birthweights of Japanese newborns were approximately 300 g lower than that of Caucasian newborns and approximately 150—170g lower than that of South Korean or Chinese newborns^37^. Based on these findings and in line with International Society of Ultrasound in Obstetrics and Gynecology (ISUOG) recommendations, the creation of local charts and comparing them with other reference charts is considered meaningful.

Fetal growth has been reported to be generally similar across geographically diverse settings, as shown in studies related to the INTERGROWTH-21st Project^38^. Although the authors concluded that ethnic differences could be disregarded, their analysis of standardized site discrepancies (SSD) revealed that HC values were approximately 0.5 SD smaller in Asian countries and approximately 0.5 SD larger in European and North American populations. These differences may be clinically significant, as the universal application of global standards could lead to overdiagnosis or underdiagnosis of growth abnormalities. Our findings, which show that fetal biometric measurements and EFW in the Japanese population are smaller than those reported in both international and other Asian studies, highlight the necessity for population-specific reference values in the assessment of fetal growth.

### Establishment of low bias reference values

While various methods have been reported to establish reference values for fetal biometric parameters, a prospective, population-based study design is recommended^17,39^. A systematic review recommended the use of a checklist and reported a positive correlation between quality score and publication year^17^. To minimize bias, this study was designed with reference to the checklist. Finally, this manuscript was prepared in accordance with the STROBE checklist^40^. The representativeness of our study population is supported by national statistics and large-scale reports from Japan. According to national data, the mean maternal age in Japan is 32.3 years, the average pre-pregnancy maternal weight is 53.1 kg, and the mean birthweight is approximately 3.0 kg^41^. These values are comparable to those observed in our study cohort. Furthermore, the prevalence of HDP and GDM in our cohort aligns with findings from previous large-scale Japanese studies^42,43^. Taken together, these findings indicate that our study cohort is reflective of the general obstetric population in Japan and supports the generalizability of our results to contemporary Japanese obstetric practice at all.

### Clinical implications

ISUOG also recommends that regional standards should be considered in fetal assessment, and indeed, the use of local data may reduce false-positives diagnosis of FGR. In line with this recommendation, the new fetal growth reference charts developed in this study are expected to improve the accuracy of fetal growth assessment in clinical practice in a Japanese population. Furthermore, these findings contribute not only to the refinement of fetal growth assessment in Asia but also to the broader goal of establishing standardized international criteria for fetal growth evaluation across diverse populations.

### Strengths and limitations

The strength of this study lies in its high-quality, prospective, multicenter design, which ensured measurement accuracy by limiting participation to facilities staffed by fellows certified by the JSUM. As a result, fetal biometric data were collected from many participants (n = 3,753) across over 50 facilities distributed throughout Japan. This wide geographical distribution enhances the generalizability of the reference charts to the Japanese population. This study has also limitation. We did not assess the external validity of the newly developed fetal biometry and estimated fetal weight (EFW) reference charts. The clinical validity and usefulness of these charts, including the cut-off values used for fetal growth assessment, should be carefully evaluated in future studies.

## Conclusions

This study successfully established high-quality, population-based reference values for fetal ultrasound biometry in Japan. Our findings demonstrated that fetal biometric measurements in the Japanese population are consistently lower than those reported in international studies, and are also smaller than those in other Asian populations.

## Supplementary Information

Supplementary Information 1.

Supplementary Information 2.

## Data Availability

The data that supports the findings of this study are available in the Table S5 (Supplementary Information 2) of this article.
